# HBV inhibits apoB production via the suppression of MTP expression

**DOI:** 10.1186/1476-511X-10-207

**Published:** 2011-11-11

**Authors:** Fu-Bing Wang, Cheng-liang Zhu, Xinghui Liu, Guo-sheng Gao

**Affiliations:** 1 Department of Clinical Laboratory, Zhongnan Hospital, Wuhan University, Wuhan, 430071, PR China; 2 Department of Clinical Laboratory, Renmin Hospital of Wuhan University, Wuhan, Wuhan 430060, PR China; 3 Department of Clinical Laboratory, Dongfeng Hospital, Hubei University of Medicine, Shiyan, 442008, PR China; 4 Department of Clinical Laboratory, Ningbo NO.2 hospital, Ningbo, 315010, PR China

**Keywords:** hepatitisB virus, chronic HBV infection;lipid metabolism, apolipoprotein B, microsomal triglyceride transfer protein

## Abstract

**Background:**

Liver dominates the production and secretion of apolipoprotein B (apoB) and evidence shows that liver malfunction induced by hepatitis B virus (HBV) infection could lead to apolipoprotein metabolism disorders. The present study was undertaken to assess the effects of HBV on apoB expression.

**Methods:**

Clinical examination: serum apoB levels in patients with chronic HBV infection and in healthy individuals were measured by immunoturbidimetry using biochemical analyzer Olympus 5400. Cell study: mRNA and protein expression levels of apoB in HepG2 and HepG2.2.15 cells were measured by RT-PCR and Western blot. Alternatively, HBV infectious clone pHBV1.3 or control plasmid pBlue-ks were tranfected into HepG2 cells, and mRNA and protein expression levels of apoB, as well as the microsomal triglyceride transfer protein (MTP) in tranfected HepG2 cells were also measured by RT-PCR and western blot.

**Results:**

Serum apoB level was much lower in chronic HBV patients as compared to healthy individuals (P < 0.05). Expression of apoB mRNA and protein was lower in HepG2.2.15 cells than in HepG2 cells. Similarly, expression of apoB mRNA and protein was lower in pHBV1.3 transfected HepG2 cells than in pBlue-ks transfected HepG2 cells. Expression of MTP mRNA and protein in pHBV1.3 transfected HepG2 cells was reduced in a dose-dependent fashion.

**Conclusion:**

HBV infection plays an inhibitory effect on apoB expression.

## Introduction

Hepatitis B virus (HBV) is a 3.2-kb, hepatotropic DNA virus that infects about one third of the world's population (2 billion) at present time. Although acute infection of HBV in most people can trigger natural immunity to this virus, patients with persistent infection are prone to developing chronic hepatitis, liver cirrhosis, and/or hepatocellular carcinoma (HCC) [1-3]. According to WHO, an estimated 600,000 people die every year due to the consequences of HBV infection [4]. It is generally believed that HBV infection is non-cytopathic, with its disease pathogenesis mediated by host innate and adaptive immune responses, as well as other host-virus interactions [5].

Liver plays an important role in lipid metabolism and is thought to be the major assembly center for the production of most endogenous lipids, apolipoproteins, and lipoproteins. Apolipoprotein B-100 (apoB), a major protein component of very low density lipoprotein (VLDL) and low density lipoprotein (LDL), is made in the liver and required for stabilizing lipoprotein structure. ApoB helps direct transport of triglycerides that is part of the VLDL or LDL complex and acts as a ligand recognized by the LDL receptor to exert effects on cholesterol metabolism. Increased plasma apoB and LDL cholesterol levels are considered as risk factors for coronary heart disease [6-8]. Another protein factor that is absolutely required for VLDL assembly and maturation is the microsomal triglyceride-transfer protein (MTP). In the absence of MTP, apoB- containing lipoprotein syntheses cannot be achieved properly. Under physiological circumstances, the integrity of cellular functions of liver ensures lipid metabolism homeostasis [9,10]. In contrast, pathological factors induced hepatic cellular dysfuntion and damage can lead to plasma lipid and lipoprotein disorders. For instance, hypertriglyceridemia and decreased plasma high density lipoprotein (HDL) are found in patients suffered from acute hepatitis B, and chronic lipid consumption and reduced serum lipid levels are often associated with the persistent HBV infection [11].

It has been demonstrated that HBV infection leads to changes of apolipoprotein mRNA abundance in cultured hepatoma cells [12]. The present study was undertaken to assess the effects of HBV on apoB expression. We measured and compared the serum apoB levels in patients with chronic HBV infection and in healthy individuals, and analyzed the apoB mRNA and protein expression levels in the presence of HBV at the cellular level. Our results suggest there is an inverse correlation between HBV infection and apoB expression.

## Materials and methods

### Study population

148 chronic hepatitis B patients (CHB) were recruited from Zhongnan Hospital of Wuhan University in this study from June 2009 through March 2011. The diagnosis of chronic hepatitis B(CHB) was confirmed by the serological examination of HBsAg for more than 6 months. 116 healthy donors negative for all viral hepatitis markers and with normal liver function profile served as controls. This study was in compliance with the Helsinki Declaration, and all patients gave written informed consent for participation.

### Measurement of apoB serum levels

Measurement of apoB serum levels was determined by immunoturbidimetry using an automated spectrophotometer (Olympus AU 5400, Olympus Optical Co., Japan) and a commercial kit (RANDOX Laboratories Ltd., United Kingdom).

### Cell culture and transfection

HepG2 and HepG2.2.15 human liver cell lines were obtained from American Type Culture Collection (A.T.C.C.), Manassas, VA, U.S.A. Both cell lines were maintained using DMEM (Dulbecco's modified Eagle's medium) supplemented with 10% fetal bovine serum at 37 °C in a humidified 5% CO2 incubator. Transient transfections of HepG2 cell with pHBV1.3 (a plasmid containing 1.3-fold HBV genome as described previously) or pBlue-ks were conducted using Lipofectin2000 (Invitrogen, U.S.A) following the manufacturer's instruction [13].

### Reverse Transcriptase (RT)-PCR analysis

Total RNA from cell samples was prepared using TRIZOL (Invitrogen, Carlsbad, CA, USA) and then reverse transcribed with an oligo(dT) primer. The resulting cDNA was PCR amplified with the gene-specific primers designed for:

ApoB, 5'-CACAGGCATCAGCCCACTT-3' (sense)

and 5'-TGCGAGGCCCATCTTCTTA-3'(antisense);

MTP, 5'-CGTTCGGCATCTACTTACA-3'(sense)

and 5'-GACCACCCTGGACCTCTAT-3'(antisense);

β-actin. 5'-ATGATATCGCCGCGCTCG-3' (sense)

and 5'-CGCTCGGTGAGGATCTTCA -3' (antisense).

The PCR products were detected on 2% agarose gel electrophoresis and visualized under UV light with ethidium bromide stain.

### Western blot analysis

Cell samples were lysed with Nonidet P-40 lysis buffer (10 mM Tris-HCl (pH 7.4), 10 mM NaCl, 3 mM MgCl_2_, and 0.5% Nonidet P-40). The cell lysates were then centrifuged at 3000 × *g *for 10 min, the supernatants were used in the assay. Protein samples were separated on 12% SDS-polyacrylamide gel electrophoresis and then transferred to nitrocellulose membrane. After blocking non-specific binding sites, western blot was performed, using specific antibody against apoB (Santa Cruz Biotechnology Inc., Santa Cruz, CA), specific antibody against MTP (Sigma), and, as an internal control, a monoclonal antibody against β-actin (Sigma). After washing, blots were developed with horseradish peroxidase-labelled goat anti-rabbit IgG, using an Enhanced Chemiluminescence Kit (Amersham Life Sciences).

### Statistical analysis

Statistical analysis was performed with SPSS13.0 software. Values were expressed as means ± SD. Means were compared by *t *tests to determine statistical significance. A *p *value less than 0.05 (*P *< 0.05) was considered significant.

## Results

### Comparison of serum apoB levels between chronic HBV patients and healthy controls

**Figure 1:**
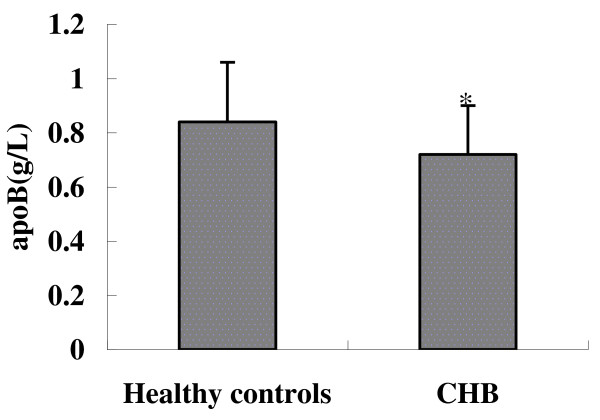
**Serum apoB levels in chronic HBV patients and healthy controls**. *p<0.05 (CHB patients *versus *healthy controls).

To investigate the correlation between HBV infection and apoB expression, we examined the serum apoB levels in CHB patients compared to HBV-negative donors. As shown in Figure 1, the serum levels of apoB was much lower in CHB as compared to healthy individuals (0.72 ± 0.18 g/L vs 0.84 ± 0.22 g/L).

### Effect of HBV on levels of apoB mRNA and protein

**Figure 2:**
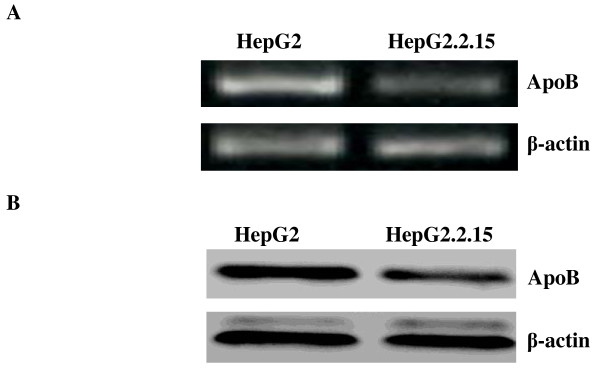
**ApoB mRNA and protein expression in HepG2 cells and HepG2.2.15 cells assessed by RT-PCR and Western blot, respectively**. (A) RT-PCR analysis was used to compare the relative levels of apoB mRNA in HepG2 cells and HepG2.2.15 cells. β-actin gene was amplified as an internal control. PCR products were detected on 2% agarose gel electrophoresis with ethidium bromide stain.(B) Western blot analysis of apoB protein expression in HepG2 cells and HepG2.2.15 cells. Protein samples from whole cell extracts were fractionated on 12% SDS-PAGE and then subjected to Western blot using a specific antibody to apoB. β-actin was the internal control.

Previous studies demonstrated that HBV infection alters the expression of a wide array of apolipoprotein genes including apoB [12]. To further analyze the effect of the HBV on apoB synthesis, the relative expression of apoB mRNA in HepG2 cells and HepG2.2.15 cells was evaluated by RT-PCR. Cellular RNA was reverse transcribed and then amplified with primers specific for apoB and housekeeping gene β-actin. As shown in Figure 2A, the expression of apoB mRNA in HepG2.2.15 cells was significantly lower compared with that in HepG2 cells. In addition, the β-actin mRNA remained constant between the two cell lines.

To determine apoB protein expression in HepG2 and HepG2.2.15 cells, Western blot analysis was performed using apoB- and β-actin- specific antibodies. As shown in Figure 2B, the expression level of apoB in HepG2 cells was much higher than that in HepG2.2.15 cells, indicating that the apoB protein levels in HepG2 and HepG2.2.15 cells closely follow the changes in mRNA levels. In addition, β-actin protein synthesis remained constant between the two cell lines.

**Figure 3:**
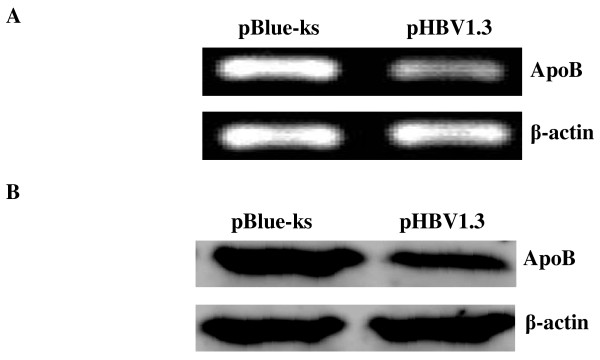
**ApoB mRNA and protein expression in HepG2 cells transfected with pHBV1.3 and HepG2 cells transfected with pBlue-ks assessed by RT-PCR and Western blot, respectively**. (A) RT-PCR analysis was used to compare the relative levels of apoB mRNA in HepG2 cells transfected with pHBV1.3 and HepG2 cells transfected with pBlue-ks. β-actin gene was amplified as an internal control. PCR products were detected on 2% agarose gel electrophoresis with ethidium bromide stain.(B) Western blot analysis of apoB protein expression in HepG2 cells transfected with pHBV1.3 and HepG2 cells transfected with pBlue-ks. Protein samples from whole cell extracts were fractionated on 12% SDS-PAGE and then subjected to Western blot using a specific antibody to apoB. β-actin was the internal control.

To rule out the possibility that the chosen of HepG2.2.15 cells coincidentally reduce apoB gene expression due to the clonal differences between HepG2 and HepG2.2.15 cells, the relative expression of apoB was reexamined in HepG2 cells transfected with a plasmid containing 1.3-fold HBV genome (pHBV1.3) and in HepG2 cells transfected with a control plasmid (pBlue-ks). Results showed that both apoB mRNA and protein levels were significantly reduced in the HBV transfected HepG2 cells, confiming the specific inhibitory effect on apoB synthesis in the presence of HBV (Figure 3).

### Effect of HBV on levels of MTP mRNA and protein

**Figure 4:**
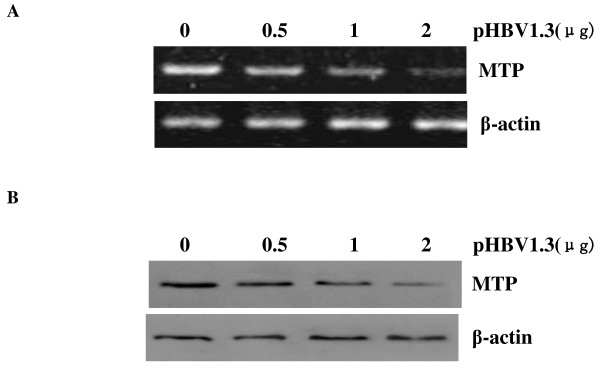
**MTP mRNA and protein expression in HepG2 cells transfected with different amounts of pHBV1.3**. (A) RT-PCR analysis was used to compare the relative levels of MTP mRNA in HepG2 cells transfected with 0 μg, 0.5 μg, 1 μg, and 2 μg pHBV1.3. β-actin gene was amplified as an internal control. PCR products were detected on 2% agarose gel electrophoresis with ethidium bromide stain.(B) Western blot analysis of MTP protein expression in HepG2 cells transfected with 0 μg, 0.5 μg, 1 μg, and 2 μg pHBV1.3. Protein samples from whole cell extracts were fractionated on 12% SDS-PAGE and then subjected to Western blot using a specific antibody to MTP. β-actin was the internal control.

Both MTP and apoB are major regulators of VLDL assembly and secretion in the liver. MTP has been shown to physically interact with apoB and play a chaperone role for apoB synthesis. To test the possibility that HBV might affect MTP expression, MTP mRNA and protein levels in the HBV transfected HepG2 cells were measured by RT-PCR and Western blot respectively. As shown in Figure 4, HepG2 cells were transfected with different amounts of pHBV1.3. Both MTP mRNA and protein levels in the HBV transfected HepG2 cells decreased in a dose-dependent fashion.

## Discussion

ApoB is the major structural protein associated with the triglyceride-rich lipoprotein VLDLs and LDLs. Each VLDL is composed of one molecule of apoB, multiple copies of other apolipoproteins, together with varied amounts of lipids (mainly triglycerides). Since the liver plays a fundamental role in lipid metabolism, alterations of liver function are correlated with modifications of circulating lipids [14,15]. Viral hepatitis is a common cause of hepatic dysfunction and increased reports have demonstrated evident changes in serum lipid, lipoprotein, and apolipoprotein patterns in patients infected with either HBV or HCV [16-18]. In this study, we first measured and compared serum apoB levels in a series of patients with HBsAg positive chronic HBV infection and in healthy individuals. Results showed that the serum apoB levels were significantly lower in chronic HBsAg carriers than in normal controls. The mechanisms by which chronic HBV infection perturbs lipid metabolism are not well understood. To extend our knowledge in this area, we then evaluated the effects of HBV on apoB mRNA and protein levels at the cellular level. We chose HepG2 cell line as an in vitro model for investigating the influence of HBV on apoB expression because HepG2 cell has long been used for studying HBV biology and HBV host-pathogen interaction and the cell line can mimic the in vivo synthesis of apolipoproteins. Results from our cell study confirmed that the reduction of apoB synthesis is associated with the effects of hepatitis B virus itself rather than other responses.

All these information raised questions on the precise role of possible key virological factors that affect apoB synthesis. Unfortunately, due to the lack of close to reality cell-culture system and animal model, it is hard for us to specify the details of the mechanism by which HBV infection alters apoB expression. However, we can get informative cues by the comparison of HBV and HCV as the relationships between HCV infection and lipid metabolism have been under intensive investigation [19-21]. Although their genome structure and molecular organization are different, HBV and HCV utilize some common strategies to invade and replicate in hepatocytes. Among them, both viruses share common routes of transmission, depend on host cell machinery to carry out viral gene expression, and are able to establish persistent infection and chronic liver diseases. A unique property of HCV is its association with lipid metabolism throughout its life cycle. The multifaceted interactions between HCV and lipid metabolism facilitate the entry and replication of virus in host cell as well as allow virus to evade immune surveillance. It has been reported that one of the HCV nonstructural proteins NS5A inhibits apoB secretion [22]. Similar to NS5A, HBV × protein is a multifuncional transactivator that has been shown to transactivate a variety of virus and host cell promoters [23]. Thus, we propose that HBx might be one of the key molecules in the regulation of apoB synthesis. Our hypothesis of the role of HBx was verified by the discovery that HBx could increase the expression of β1,4-N-acetylglucosaminyltransferase III (GnT-III), which induced aberrant glycosylation of apoB and disrupted apoB secretion [24]. To better understand the regulation of apoB gene expression, the cis-acting elements on the apoB promoter and the putative transacting factors which interact with these elements have been defined. Therefore, development of tissue culture systems that support replication of HBV and production of infectious virions is quite urgent so as to permit a more complete understanding of the molecular basis of the interactions between HBV and lipid metabolism.

Our studies further revealed that MTP synthesis was decreased in HBV transfected HepG2 cells. This result provides another reasonable explanation to the HBV-induced apoB reduction. MTP is an intracellular lipid transfer protein that catalyzes the transport of triglyceride, cholesteryl ester, and phospholipid molecules between membranes. It is a heterodimer of two proteins of molecule weight 97 kD and 58 kD, both of which are required for functional activity. MTP plays an essential role in the cotranslocational lipidation of apoB as newly synthesized apoB is translocated into the lumen of the endoplasmic reticulum (ER), MTP can also binds directly to the nascent apoB to play a chaperone role in directing apoB into the ER lumen. Hepatic overexpression of MTP results in increased in vivo secretion of VLDL triglycerides and apoB [25,26].

In addition to functioning primarily as an energy reserve and as building material of the cell membrane of almost all living organisms, lipids participate in the host immune response to infections. It appears that triglyceride-rich lipoproteins such as VLDL can inactivate a variety of infectious agents, play an immune regulatory role, and are involved in the maintenance of immunologic homeostasis [27]. Actually, lipid metabolism and immune response are tightly integrated and the proper function of each is dependent on the other. The inhibitory effects of HBV on apoB and MTP expression suggest that HBV may interfere with VLDL assembly and/or secretion, which would represent an unusual virus-host interaction that favors evasion of the host antiviral immune response.

In summary, the results of our clinical examination and cell study demonstrate the ability of HBV to disturb apoB expression, which may provide practical information contributing to the diagnosis of liver pathology and effective therapies for HBV infection.

## Competing interests

The authors declare that they have no competing Interests.

## Authors' contributions

FBW participated in the measurement of apoB serum levels, Cell culture and transfection. GSG performed reverse Transcriptase (RT)-PCR analysis and western blot. XHL participated in the statistical analysis. CLZ participated in its design. All authors read and approved the final manuscript.
